# The Relationship Between Transition Shock and Nursing Informatics Competency in Nursing Interns: A Moderated Mediation Model of Psychological Empowerment and Teaching Hospital Grade

**DOI:** 10.1155/jonm/9706679

**Published:** 2026-09-27

**Authors:** Miao Wang, Xinle Zhou, Tong Wu, Xudong Fan, Qing Pan, Hui Guan

**Affiliations:** ^1^ School of Nursing, Wannan Medical Universtiy, Wuhu, Anhui, China; ^2^ Department of Nursing, West Anhui Health Vocational College, Lu’an, Anhui, China

**Keywords:** nursing informatics competency, nursing interns, psychological empowerment, teaching hospital, transition shock

## Abstract

**Background:**

From academic study at school to clinical practice, changes in the environment, social role, and learning objectives can lead student nurses to experience varying degrees of transition shock. In today’s digitally driven healthcare environment, where information literacy is a core competency, this transition shock may seriously hinder interns’ nursing informatics competency. However, the exact mechanism by which transition shock impacts the interns’ nursing informatics competency remains underexplored.

**Aim:**

To explore the effects of transition shock on the nursing informatics competency of interns, as well as the roles of psychological empowerment and teaching hospital grade.

**Methods:**

A total of 450 participants were recruited from an undergraduate medical college and a health vocational college in Anhui Province, eastern China, between October and December 2025 in this cross‐sectional study. Data collection instruments included the General Information Questionnaire, the Nursing Informatics Competency Scale for Undergraduate Nursing Students, the Psychological Empowerment Scale, and the Clinical Practicum Transition Shock Scale. Moderated mediation analysis was performed using the PROCESS Macro for SPSS 26.0.

**Results:**

A total of 435 valid questionnaires were included in this study. Participants′ nursing informatics competency was moderate to high. Transition shock did not directly affect interns’ nursing informatics competency (*β* = −0.014, *p* > 0.05). However, psychological empowerment played a complete mediating role between transition shock and nursing informatics competency (95% CI: −0.275, −0.152), with a mediating effect size of −0.211. Moreover, teaching hospital grade played a moderating role in the first half of the aforementioned mediation model pathway (*β* = 0.354, *t* = 2.783, *p* < 0.01). Specifically, the mediating effect of psychological empowerment in this pathway was significant for nursing interns in both tertiary‐A hospitals (effect value = −0.153, *p* < 0.001) and non‐tertiary‐A hospitals (effect value = −0.332, *p* < 0.001).

**Conclusions:**

Psychological empowerment fully mediates the impact of transition shock on nursing informatics competency among interns. Tertiary‐A hospitals mitigate the negative effects of transition shock on psychological empowerment.

**Implications for Nursing Management:**

Nurse managers should prioritize psychological empowerment interventions for nursing interns, particularly in non‐tertiary‐A hospitals, to counteract the indirect negative association between transition shock and nursing informatics competency.

## 1. Introduction

The widespread adoption of medical information products, including hospital information systems (HIS), electronic health records, and telemedicine services, has fundamentally changed the way nursing professionals work and the competencies they require [1, 2]. Nursing informatics competency is defined as an integrated capability that combines computer literacy, information literacy, and information management technology [3]. This competency has become essential for nursing staff to ensure patient safety and improve the quality of care. Research has shown that nurses with strong nursing informatics competency can use information technology to effectively minimize task duplication and nursing errors, thus improving the quality of clinical care and safeguarding patient safety [4, 5]. Clinical placement is a critical phase in the transition of nursing students into professional nurses. For nursing interns still in the clinical learning phase, mastering and improving nursing informatics competency in complex healthcare settings are important for successfully transitioning into professional roles and developing their careers. However, studies have indicated that the overall level of nursing informatics competence among nursing students remains moderate to low globally. A systematic review of 13 studies reported that nursing students had an average total nursing informatics competency score of 3.4 (on a 5‐point scale), with considerable variation across different competency dimensions [6]. In China, recent studies have shown that undergraduate nursing students score below the expected level in nursing informatics competency. A survey of 1715 nursing interns reported a mean score of 2.87 out of 5, which indicates that current educational strategies may not adequately prepare students to meet the technological requirements of clinical practice [7, 8]. This situation can largely be attributed to the early‐stage development of nursing informatics education in China: informatics courses have not yet been comprehensively and systematically integrated into nursing licensure preparation programs, which makes clinical placement a critical avenue for nursing interns to develop their nursing informatics competencies [9, 10]. Therefore, investigating the factors influencing nursing informatics competency among nursing interns and their underlying mechanisms is necessary to enhance it. Nevertheless, the key factors that facilitate or impede the development of nursing informatics competency among nursing interns remain largely unexplored.

Transition shock refers to the feelings of confusion, disorientation, doubt, and uncertainty that individuals experience when transitioning from a familiar role to an unfamiliar one [11]. It is usually influenced by factors such as the role itself, relationships, knowledge, and responsibilities. During the transition from student to registered nurse, nursing interns often experience varying degrees of transition shock due to complex interpersonal adaptation, increased clinical accountability, underdeveloped clinical skills, and a gap between theoretical knowledge and practical demands [12, 13]. Studies have shown that Chinese nursing interns experience moderate to high levels of clinical transition shock [14, 15]. Such elevated transition shock not only undermines nursing interns′ professional identity and professional self‐concept but also impairs their attitudes toward patient safety [13, 15]. Research also confirms that transition shock is significantly positively associated with the occurrence of adverse patient events [16]. Regarding competency development, existing empirical evidence indicates that transition shock is significantly and negatively correlated with the overall clinical competence of new nurses and identified emotional challenges as a key predictor of professional competence [17, 18]. A Chinese study of 1172 new nurses in tertiary general hospitals further confirmed that transition shock is significantly negatively correlated with nursing informatics competency (*r* = −0.491, *p* < 0.05) [19]. However, empirical research directly linking transition shock to nursing informatics competency among nursing interns remains scarce. A recent study conducted among Chinese nursing interns found that transition shock directly predicts mental health outcomes (effect size = 0.417), and nursing information ability mediates this relationship (effect size = 0.381), indicating a potential association between transition shock and informatics competency–related outcomes [20]. Limited evidence further suggests that concerns about operational errors related to transition shock may lead nursing interns to avoid using nursing information systems, hindering their in‐depth learning and practical engagement with clinical information technology [21]. Based on these theoretical and empirical considerations, this study proposes Hypothesis 1: clinical transition shock negatively predicts interns′ nursing informatics competency.

To systematically examine the mechanisms underlying the relationship between transition shock and nursing informatics competency, this study draws on Conservation of Resources (COR) theory [22, 23]. COR theory posits that individuals are motivated to acquire, retain, and protect their valued resources, which include objects, personal characteristics, conditions, and energy. It also holds that resource loss has a disproportionately greater psychological impact on individuals than resource gain [22, 23]. When individuals encounter stressful events that threaten or deplete their key internal resources, they will adopt defensive strategies to prevent further resource loss, which may manifest as adaptive withdrawal, reduced initiative, and weakened learning behavior [24, 25]. In the context of this study, clinical transition shock acts as a resource‐depleting stressor that consumes the psychological and emotional resources of nursing interns.

Psychological empowerment refers to an individual’s internal cognitive state and psychological condition, characterized by a sense of meaning, competence, autonomy, and influence experienced in the workplace [26]. It is recognized as a critical internal personal resource that can stimulate individuals’ intrinsic motivation at work and improve their work behavior, attitudes, and outcomes [27, 28]. However, high‐intensity transition shock may undermine nursing interns′ perceived control, sense of meaningfulness, and self‐efficacy [29]. Drawing on COR theory, transition shock, as a resource‐depleting stressor, directly erodes available internal resources (psychological empowerment), thereby impairing individuals’ capacity for proactive learning and skill acquisition (nursing informatics competence). This framework supports positioning psychological empowerment as a mediator: the resource depletion triggered by transition shock is hypothesized to erode psychological empowerment, subsequently hindering the development of nursing informatics competence. Accordingly, Hypothesis 2 is proposed: Psychological empowerment mediates the relationship between transition shock and nursing informatics competency among nursing interns.

However, psychological empowerment is not merely an intrinsic trait but a malleable cognitive construct shaped by organizational environments and working conditions [30]. While COR theory provides a solid theoretical framework for understanding the mediating role of psychological empowerment, the degree to which transition shock depletes psychological resources varies substantially among nursing interns, partially depending on their respective organizational and environmental contexts. This contextual variability is particularly distinct in the Chinese nursing education system. Unlike the dispersed clinical placements adopted in most Western countries, major Chinese nursing education programs require students to complete a centralized, full‐time internship in their final academic year, which generally lasts 40–48 weeks. During the internship, nursing interns undertake most core patient care tasks under the guidance and supervision of registered nurses. Although their work autonomy remains limited, the immersive nature of these clinical rotations exposes them to workload intensities and stressors that closely match those faced by entry‐level licensed nurses [31].

Given this immersive working experience, organizational context and resource availability exert a critical influence on nursing interns′ psychological adaptation and competency development. In China, hospital grade serves as a comprehensive indicator of the quality of a hospital’s organizational environment and resource endowment. Under China’s national three‐tier, 10‐level medical institution classification system, tertiary‐A hospitals stand at the top of the healthcare hierarchy. These institutions have abundant medical resources, advanced technological infrastructure, and robust teaching systems, but they also handle much larger patient volumes and treat clinically more complex cases [32, 33]. This structural duality creates a complex organizational environment for nursing interns, characterized by the interplay of opportunities and challenges. On one hand, abundant external resources—including structured training programs, supportive learning climates, and accessible mentorship—help buffer the psychological distress caused by transition shock. On the other hand, core characteristics of these institutions, such as high patient turnover, clinical complexity, and intense interpersonal dynamics, impose exceptionally high job demands that may accelerate the depletion of psychological resources [14]. Empirical evidence consistently shows that demanding work environments can negatively impact the development of novice practitioners′ psychological resource reserves and professional competence [34, 35].

From a COR perspective, the concurrent presence of abundant resources and high job demands creates two competing mechanisms: abundant resources may buffer against psychological resource loss; however, excessive job demands may intensify it. Since nursing interns have limited initial psychological reserves, they are particularly sensitive to which of these opposing forces dominates. Consequently, the strength of the negative association between transition shock and psychological empowerment among nursing interns may vary across different hospital grades. A key understudied empirical question is whether the resource‐rich environment of tertiary‐A hospitals buffers nursing interns against transition shock and thereby weakens this negative association, or whether the high‐demand characteristics of these settings exacerbate resource depletion and thereby strengthen this association. This research question is particularly relevant against the backdrop of China’s unique combination of a hierarchical hospital system, centralized nursing internship model, and rapid healthcare digitalization. This context has so far drawn little empirical attention. Accordingly, we propose Hypothesis 3: Hospital grade moderates the relationship between clinical transition shock and psychological empowerment, such that this association is either attenuated (or amplified in tertiary‐A hospitals compared with non‐tertiary‐A hospitals).

In summary, grounded in COR theory, this study proposes a moderated mediation model (see Figure 1): psychological empowerment mediates the relationship between transition shock and nursing informatics competency among nursing interns, while hospital grade moderates this mediating pathway by influencing the resource loss process from transition shock to psychological empowerment. Specifically, this study aims to examine whether the indirect effect of transition shock on nursing informatics competency via psychological empowerment is moderated by hospital grade (tertiary‐A vs. non‐tertiary‐A). We expect the findings to provide localized empirical evidence to identify key barriers to nursing informatics competency development across different hospital grades, to develop tier‐specific clinical teaching support strategies, and to extend the applicability of COR theory to nursing education in the Chinese context.

**FIGURE 1:**
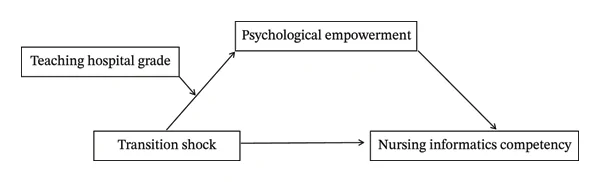
The hypothesized moderated mediation model.

## 2. Methods

### 2.1. Study Design and Setting

This study adopted a cross‐sectional design and was reported in accordance with the STROBE guidelines. The survey was conducted at a 3‐year health vocational college and a 4‐year undergraduate medical college in Anhui Province, China. These two nursing schools are affiliated with 46 clinical teaching hospitals, distributed across multiple cities: Hefei, Wuhu, Huangshan, Lu’an, Fuyang and Bengbu in Anhui Province; Nanjing, Xuzhou and Yixing in Jiangsu Province; Hangzhou in Zhejiang Province; and Shanghai City. Among these 46 hospitals, 36 are tertiary‐A hospitals and 10 are non‐tertiary‐A hospitals. As the original study design only required participants to report the grade of their internship hospital, without recording the specific hospital name, we are unable to provide the exact number of hospitals at each tier. Nevertheless, the range described above reflects the basic sampling framework of this study. The inclusion criteria were as follows: (1) nursing students currently undertaking clinical internships, (2) aged over 18 years, and (3) fully informed of the study details, provided consent and participated voluntarily. The exclusion criteria were (1) nursing students who had been absent from clinical placement for more than 2 weeks for any reason; (2) participants with health conditions that could affect the validity of the results, such as depression or severe physical illness.

### 2.2. Sample Size

The sample size was determined using G∗Power 3.1.9.7. A linear multiple regression (fixed model, *R* ^2^ increase test) was selected [36]. Based on Cohen’s conventions and findings from similar studies [37], an effect size of *f* ^2^ = 0.02 (a small effect) was adopted. The analysis indicated a minimum required sample size of 395 participants. With an anticipated invalid response rate and 10% attrition, the aim was to recruit 439 nursing interns. Finally, 450 nursing interns were recruited for this study.

### 2.3. Measures

#### 2.3.1. General Information Questionnaire

The questionnaire consists of 8 items, including variables such as age, sex, whether one is an only child in the family, whether one is a class leader, educational programs, whether one has completed a nursing informatics course in school, length of completed clinical placement, and teaching hospital grade.

#### 2.3.2. Nursing Informatics Competency

The Nursing Informatics Competency Scale for Undergraduate Nursing Students (NICSUNS) was developed by Le et al. [38] to evaluate the nursing informatics competency of undergraduate nursing students in China. It consists of five dimensions, including 36 items: basic computer competency (7 items), information knowledge and skills (11 items), information management competency (6 items), information law and safety (6 items), and information attitude (6 items). Responses are rated on a 5‐point Likert scale, with scores ranging from 36 to 180, and higher scores indicate stronger nursing informatics competency. The Scale‐level Content Validity Index (S‐CVI) was 0.96, the split‐half reliability was 0.901, the overall Cronbach’s *α* coefficient was 0.97, and Cronbach’s *α* for its subscales ranged from 0.90 to 0.94. The test–retest reliability was 0.82. The overall Cronbach’s *α* coefficient in this study was 0.96.

#### 2.3.3. Transition Shock

The Clinical Practicum Transition Shock Scale (CPT‐Shock) was originally developed by Kim and Ko [39] to evaluate the level of transition shock experienced by nursing interns during their clinical practice. It was subsequently translated and adapted into Chinese by Lyu et al. [40]. The Chinese version of CPT⁃Shock comprises 6 dimensions, including unfriendly interpersonal relationships (4 items), disparity between theory and nursing practice (4 items), pressure felt in real situations (3 items), changes in daily life patterns (3 items), worries about becoming a clinical nurse (3 items), and role identity confusion (2 items), with a total of 19 items. Responses are rated on a 4‐point Likert scale. Total scores range from 19 to 76, with higher scores indicating stronger transition shock. The Chinese version of the CPT‐Shock showed solid psychometric properties. The CVI for the scale was 0.95, the Cronbach’s *α* coefficient was 0.86, and Cronbach’s *α* for its subscales ranged from 0.76 to 0.83. Exploratory factor analysis (EFA) supported its dimensional structure, explaining 71.83% of the variance; the Kaiser–Meyer–Olkin (KMO) value was 0.79. Confirmatory factor analysis (CFA) indicated a satisfactory model fit (*χ* ^2^/df = 1.58, CFI = 0.94, IFI = 0.94, TLI = 0.92, RMSEA = 0.05). In this study, Cronbach’s *α* coefficient for the scale was 0.94.

#### 2.3.4. Psychological Empowerment

The Psychological Empowerment Scale (PES) was first developed by Spreitzer [27]. It was modified by Li et al. [41] for use in a Chinese cultural context. The Chinese version of the PES had 4 dimensions and 12 items: meaning (3 items), self‐determination (3 items), competence (3 items), and impact (3 items). Respondents rated their agreement with each item on a five‐point Likert scale from 1 (strongly disagree) to 5 (strongly agree). The total score ranges from 12 to 60. A higher score indicates greater psychological empowerment. Cronbach’s *α* for its subscales ranged from 0.72 to 0.86. The factor structure was well supported: EFA explained 73.78% of the variance, and CFA demonstrated a satisfactory fit (*χ* ^2^/df = 2.22, CFI = 0.98, IFI = 0.98, TLI = 0.97, RMSEA = 0.04). In our study, Cronbach’s *α* coefficient for the scale was 0.88.

### 2.4. Data Collection

The Wenjuanxing platform (https://www.wjx.cn/) was used to create the electronic questionnaire and generate a dedicated QR code and questionnaire link. During the online class meeting, the researcher issued the recruitment notice and shared the QR code and link in the class online community. Interested nursing interns could use mobile devices such as smartphones or tablets to scan the QR code or click the link to access the questionnaire page. To protect participants′ privacy and improve convenience, the questionnaire was completed and submitted anonymously online. Only one submission is permitted per device or IP address to prevent duplicate responses. We also set up immediate alerts for outliers in numerical questions. For example, participants would receive the following prompt: “The age you entered appears to be too young. Please verify the accuracy of your entry.” Finally, we excluded invalid questionnaires according to pre‐established criteria and conducted further data cleaning to identify and eliminate any remaining outliers or errors.

### 2.5. Data Analysis

The data were processed using SPSS 26.0 software. Firstly, Harman’s single‐factor test was used to examine common method bias. Normality of continuous variables was assessed using the Kolmogorov–Smirnov test, complemented by visual inspection of Q–Q plots and evaluation of skewness and kurtosis coefficients. Means and standard deviations were used to describe continuous variables, and frequencies and percentages were used to describe categorical variables. Independent samples *t*‐tests or one‐way ANOVA were used for group comparisons, and Pearson correlation analysis was conducted to examine bivariate correlations among the core variables. Furthermore, Model 4 in SPSS PROCESS [42] was used to evaluate the mediating effect of psychological empowerment in the relationship between transition shock and nursing informatics competency. Finally, Model 7 in PROCESS was employed to test the moderating role of teaching hospital grade in the relationship between transition shock and psychological empowerment. Before inclusion in the model analysis, all continuous variables were standardized using the *Z*‐score method to facilitate subsequent comparisons across different measurement scales. The significance level was set at *α* = 0.05.

### 2.6. Ethical Considerations

The study strictly adhered to local regulatory requirements, applicable laws, and the ethical standards set out in the Declaration of Helsinki. The protection of participants’ rights and welfare was prioritized throughout the research process. The recruitment notice emphasized that participation in the survey was voluntary and that data would be collected anonymously. The introductory section of the electronic questionnaire provided detailed information about the study, including its objectives, content, data storage, and usage. Interested nursing interns could choose whether or not to participate. Completing and submitting the questionnaire were considered informed consent to participate in the research. Participants could withdraw at any time during the process without facing any negative consequences. Ethical approval for this study was obtained from the Research Ethics Committee of the School of Nursing at Wannan Medical College, China (NO. 20250010).

## 3. Results

### 3.1. Descriptive Statistics and Common Method Bias Test

This study analyzed 435 valid responses, resulting in an effective response rate of 96.67%. A total of 15 questionnaires were excluded: Nine had entirely identical responses, and six were completed in under 60 s, indicating inattentive responding. The sociodemographic characteristics of the sample are shown in Table 1. The mean age was 21.15 years (SD = 1.16), and the sample was predominantly female (79.77%). Most participants were not the only child in their family (70.34%) and were enrolled in a 4‐year undergraduate nursing program (70.57%). Just over half of the participants reported having completed a formal nursing informatics course during their academic training (51.95%). Regarding clinical placement experience, the majority had completed 3 months or less of clinical practice (58.85%). Finally, 340 participants (78.2%) were completing their clinical placement at tertiary‐A hospitals, while the remaining 95 participants (21.8%) were placed at non‐tertiary‐A hospitals. The results of Harman’s single‐factor test revealed that the largest factor explained 27.59% of the variance, less than the recommended 40% criterion, indicating the absence of significant common method bias.

**TABLE 1:** Comparison of nursing informatics competency among nursing interns with different characteristics (*n* = 435).

Variable	*n* (%)	Score (mean ± SD)	*t/F*	*p*
Gender			1.820^a^	0.070
Male	88 (20.23)	132.17 ± 20.58		
Female	347 (79.77)	127.69 ± 20.65		
Whether an only child			−0.065^a^	0.948
Yes	129 (29.66)	128.50 ± 22.28		
No	306 (70.34)	128.64 ± 20.02		
Whether class leader			0.962^a^	0.337
Yes	146	129.94 ± 20.11		
No	289	127.92 ± 20.98		
Educational programs			−2.836^a^	0.005
3‐year junior program	128 (29.43)	124.27 ± 19.56		
4‐year undergraduate program	307 (70.57)	130.40 ± 20.91		
Whether completing a nursing informatics course in school			1.736^a^	0.083
Yes	226 (51.95)	130.25 ± 20.38		
No	209 (48.05)	126.81 ± 20.93		
Length of completed clinical placement			1.597^b^	0.204
≤ 3 months	256 (58.85)	127.93 ± 20.97		
4–8 months	92 (21.15)	127.14 ± 20.11		
≥ 9 months	87 (20.00)	132.08 ± 20.32		
Teaching hospital grade			2.939^a^	0.003
Non‐tertiary‐A hospital	95 (21.84)	134.06 ± 19.79		
Tertiary‐A hospital	340 (78.16)	127.07 ± 20.71		

^a^ *t*‐value.

^b^ *F*‐value.

### 3.2. Informatics Competency Among Nursing Interns With Different Characteristics

The Kolmogorov–Smirnov test indicated that all three variables deviated significantly from a normal distribution (*p* < 0.001). However, visual inspection of the Q–Q plots showed that the data points lay close to the diagonal line. Moreover, the absolute values of both skewness (range: 0.088–0.693) and kurtosis (range: 0.018–0.859) were well below the recommended threshold of 2.0. Taken together, these findings confirm that the data approximately satisfy the normality assumption.

Statistically significant differences in information competency were observed among nursing interns in different academic programs and among those undertaking placements in teaching hospitals of different grades (*p* < 0.05) (Table 1).

### 3.3. Correlation Analysis of the Research Variables

The nursing interns′ overall informatics competence level was 128.60 (SD = 20.69), representing 71.44% (SD = 11.49) of the maximum achievable score. Transition shock scores averaged 41.30 (SD = 11.59), equivalent to 54.34% (SD = 15.24) of the total possible, while psychological empowerment scores averaged 40.62 (SD = 7.16), amounting to 67.70% (SD = 11.93). Nursing informatics competency was negatively correlated with transition shock (*r* = −0.205, *p* < 0.01) and positively correlated with psychological empowerment (*r* = 0.506, *p* < 0.01). Transition shock was negatively related to psychological empowerment (*r* = −0.415, *p* < 0.01), as shown in Table 2.

**TABLE 2:** Pearson correlation coefficient of the variables (*n* = 435).

Variables	Mean ± SD	Mean % score ± SD	1	2	3
1. Nursing informatics competency	128.60 ± 20.69	71.44 ± 11.49	1		
2. Transition shock	41.30 ± 11.59	54.34 ± 15.24	−0.205^∗∗^	1	
3. Psychological empowerment	40.62 ± 7.16	67.70 ± 11.93	0.506^∗∗^	−0.415^∗∗^	1

^∗∗^ *p* < 0.01.

### 3.4. Mediating Role of Psychological Empowerment

Table 1 shows differences in information competency among nursing interns from different academic programs. Consequently, the academic program was incorporated as a control variable in the model analysis to examine the mediating effect. The mediation model analysis was completed using Model 4 from the SPSS PROCESS 4.2 developed by Hayes [42].

The testing of the mediating effect of psychological empowerment between transition shock and nursing informatics competency is presented in Table 3. Equation 1 indicated that transition shock harmed nursing informatics competency (*β* = −0.225, *p* < 0.001). Equation 2 showed that transition shock negatively influenced psychological empowerment (*β* = −0.417, *p* < 0.001). However, when psychological empowerment was included in the regression analysis in Equation 3, the effect of transition shock on nursing informatics competency became nonsignificant (*β* = −0.014, *p* > 0.05).

**TABLE 3:** Mediating effect of psychological empowerment between transition shock and nursing informatics competency (*n* = 435).

Variable and equation		Overall fit index			Significance of the regression coefficient	
Dependent variable	Independent variable	*R*	*R* ^2^	*F*	*β*	*t*
*Equation 1*						
Nursing informatics competency	Transition shock	0.261	0.068	15.808^∗∗∗^	−0.225	−4.813^∗∗∗^
	Academic program				0.358	3.489^∗∗∗^

*Equation 2*						
Psychological empowerment	Transition shock	0.415	0.172	44.941^∗∗∗^	−0.417	−9.443^∗∗∗^
	Academic program				0.033	0.346

*Equation 3*						
Nursing informatics competency	Transition shock	0.529	0.280	55.924^∗∗∗^	−0.014	−0.319
	Psychological empowerment				0.506	11.267^∗∗∗^
	Academic program				0.341	3.777^∗∗∗^

^∗∗∗^ *p* < 0.001.

Furthermore, the bias‐corrected percentile bootstrap method was employed with 5000 repeated samples to calculate the 95% confidence interval and test the mediating effect of psychological empowerment in the relationship between transition shock and nursing informatics competency. The results are presented in Table 4. The confidence interval (−0.103, 0.074) for the direct effect of transition shock on nursing informatics competency included zero, indicating that the direct effect was not significant. Therefore, hypothesis 1 was not supported. In contrast, the indirect impact of transition shock on nursing informatics competency via psychological empowerment was −0.211, and its confidence interval (−0.275, −0.152) did not include zero. This confirmed that the mediating effect was significant. Psychological empowerment thus fully mediated the relationship, accounting for approximately 93.8% of the total effect. Consequently, psychological empowerment fully mediated the impact of transition shock on nursing informatics competency, thereby supporting hypothesis 2.

**TABLE 4:** Bootstrap analysis of significance tests for mediating effects (*n* = 435).

Effect relationship and path	Effect value	Standard error	Bootstrap 95% CI	
			Lower limit	Upper limit
Total effect (Transition shock ⟶ Nursing informatics competency)	−0.225	0.047	−0.317	−0.133
Direct effect (Transition shock ⟶ Nursing informatics competency)	−0.014	0.045	−0.103	0.074
Indirect effect (Transition shock ⟶ Psychological empowerment ⟶ Nursing informatics competency)	−0.211	0.031	−0.275	−0.152

### 3.5. Moderating Role of Teaching Hospital Grade

A moderated mediation analysis was conducted using PROCESS Model 7 to test whether teaching hospital grade moderated the first stage of the mediation path while controlling for the academic program. Teaching hospital grade was dummy‐coded with 0 = non‐tertiary‐A hospitals and 1 = tertiary‐A hospitals.

As shown in Table 5, the interaction between transition shock and teaching hospital grade on psychological empowerment was significant (*β = 0.354, t = 2.783,* *p* < 0.01), indicating that the negative effect of transition shock on psychological empowerment was significantly weaker in tertiary‐A hospitals than in non‐tertiary‐A hospitals. Hypothesis 3 was therefore supported.

**TABLE 5:** Analysis of the moderating role of teaching hospital grade in the transition shock and psychological empowerment (*n* = 435).

Regression equation		Fitting index		Regression coefficient		
Dependent variable	Independent variable	*R* ^2^	*F*	*β*	SE	*t*
Nursing informatics competency	Transition shock	0.280	55.924^∗∗∗^	−0.014	0.045	−0.319
	Psychological empowerment			0.506	0.045	11.267^∗∗∗^
	Academic program			0.341	0.090	3.777^∗∗∗^

Psychological empowerment	Transition shock	0.191	25.336^∗∗∗^	−0.657	0.114	−5.784^∗∗∗^
	Teaching hospital grade			0.084	0.157	0.534
	Transition shock × Teaching hospital grade			0.354	0.127	2.783^∗∗^
	Academic program			0.013	0.096	0.137

^∗∗^ *p* < 0.01.

^∗∗∗^ *p* < 0.001.

To explore the moderating effect of teaching hospital grade on the relationship between transition shock and psychological empowerment, simple slope tests were conducted to examine the conditional effects of transition shock on psychological empowerment across tertiary‐A and non‐tertiary‐A hospitals. Meanwhile, transition shock was divided into high‐ and low‐level groups by mean ± 1 standard deviation, and an interaction graph was plotted accordingly (Figure 2). The results indicate that transition shock was negatively associated with psychological empowerment in both non‐tertiary‐A hospitals (*B* = −0.657, *p* < 0.001) and tertiary‐A hospitals (*B* = −0.302, *p* < 0.001). However, the negative association was significantly stronger among nursing interns in non‐tertiary‐A hospitals, suggesting that the clinical learning environment of tertiary‐A hospitals played a “buffering” role in this relationship.

**FIGURE 2:**
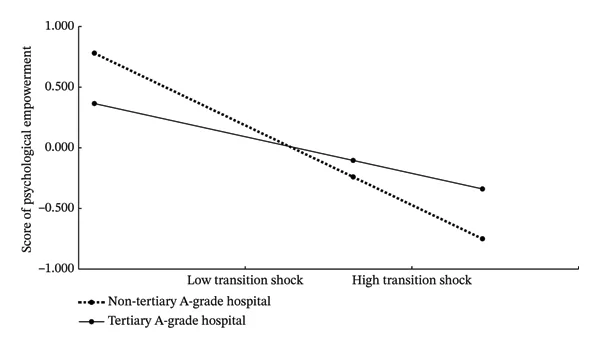
Simple slopes for tertiary‐A versus non‐tertiary‐A hospitals. The negative association between transition shock and psychological empowerment was significantly weaker in tertiary‐A hospitals (*B* = −0.302, *p* < 0.001) than in non‐tertiary‐A hospitals (*B* = −0.657, *p* < 0.001).

It was further examined whether the teaching hospital grades moderated the mediating role of psychological empowerment between transition shock and nursing informatics competency. For nursing interns in both tertiary‐A and non‐tertiary‐A hospitals, the indirect effect of transition shock on nursing informatics competency through psychological empowerment was significant. The effect sizes were −0.153 (−0.236, −0.078) and −0.332 (−0.545, −0.179), respectively, with 95% confidence intervals that excluded zero. The effect size for the two groups (0.179) indicated a significant difference, with a 95% confidence interval of (0.021, 0.396) as determined by bootstrap analysis. This suggests that transition shock has a more detrimental effect on psychological empowerment among nursing interns in non‐tertiary‐A hospitals than in tertiary‐A hospitals, with an effect strength approximately 2.17 times greater.

To further address the potential confounding effect of teaching hospital grade on informatics competency, we conducted an additional linear regression analysis with nursing informatics competency as the dependent variable, entering transition shock, psychological empowerment, teaching hospital grade, and educational program as simultaneous predictors. The results showed that psychological empowerment (*β* = 0.507, *p* < 0.001) and educational program (*β* = 0.157, *p* < 0.001) were significant predictors, while the main effects of transition shock (*β* = −0.022, *p* = 0.679) and teaching hospital grade (*β* = 0.014, *p* = 0.780) were not significant. These findings indicate that the core conclusions remain robust after controlling for hospital grade.

## 4. Discussion

The results of this study show that psychological empowerment plays a full mediating role between transition shock and nursing informatics competency among nursing interns, and the negative association between transition shock and psychological empowerment is significantly stronger in non‐tertiary‐A teaching hospitals. These findings deepen our understanding of the psychological mechanisms linking transition shock to nursing informatics competency and offer a new integrated perspective on the development of nursing informatics competency among nursing interns.

In this study, the average nursing informatics competency score of nursing interns was 128.60 ± 20.69, indicating an overall upper‐intermediate level. This result was slightly lower than the score of (132.43 ± 21.30) reported in Le’s study [39]. This discrepancy is potentially attributable to differences in sample composition: Le’s study exclusively included 4‐year undergraduate nursing students, while the participants in the current study consisted of both 4‐year undergraduate nursing interns and 3‐year diploma nursing interns. Univariate analysis confirmed that nursing informatics competency differs significantly among nursing interns from different educational programs. Consistent with previous findings [43, 44], our results show that 4‐year undergraduate nursing interns have significantly higher nursing informatics competency than 3‐year diploma nursing interns. Undergraduate nursing programs typically have a more comprehensive curriculum, often offer specialized nursing informatics courses, and place greater emphasis on developing students’ critical thinking and independent learning abilities—all of which help improve nursing informatics competency [4, 45]. By contrast, shorter diploma programs usually prioritize clinical practice skills, which may lead to a weaker foundation in nursing informatics for their students. Notably, although univariate analysis showed that nursing interns at non‐tertiary‐A hospitals had significantly higher nursing informatics competency scores than their counterparts at tertiary‐A hospitals, further multiple linear regression analysis revealed that after controlling for transition shock, psychological empowerment, and academic program, the main effect of teaching hospital grade on nursing informatics competency was not significant, while psychological empowerment and academic program remained significant predictors. Therefore, the differences observed in Table 1 are largely attributable to variations in academic program composition and psychological empowerment levels across hospital tiers, rather than a direct linear effect of hospital grade itself on informatics competency. This further indicates that in the present study, hospital grade is more appropriately conceptualized as a contextual boundary condition (i.e., a moderator) rather than a direct determinant. These findings confirm that nursing informatics competency is closely associated with educational background, and teaching hospital grade is better interpreted as a moderating contextual factor that shapes the relationship between transition shock and psychological empowerment, instead of acting as a direct predictor. Accordingly, training in nursing informatics knowledge and skills should be made more systematic during the preclinical academic phase. It is recommended that nursing informatics be set as a compulsory course module for 4‐year undergraduate nursing programs and an optional module for 3‐year diploma nursing programs. Research by Khezri and Abdekhoda [46] has confirmed that nurses’ informatics competency is positively correlated with the length of time they spend using HIS (*r* = 0.16, *p* = 0.01). Therefore, during clinical internships, teaching hospitals should increase the frequency and duration of nursing interns’ hands‐on practice with HIS to strengthen their practical operational skills and effectively improve their nursing informatics competency.

This study found that the negative association between transition shock and nursing informatics competency was fully mediated by psychological empowerment, suggesting that transition shock may undermine informatics competency primarily by depletion of interns′ psychological empowerment. This discovery provides a powerful supplement and refinement to the existing understanding of the mediating role of psychological empowerment. Previous studies mainly explored how positive organizational factors (such as professional practice environment and innovative culture) could improve nurses′ work outcomes and behavioral performance by enhancing psychological empowerment [47, 48]. This study revealed another crucial pathway of the same mechanism: negative stressors (transition shock) erode psychological empowerment, thereby hindering the development of professional learning outcomes (informatics competence). These two sets of evidence together establish the central hub role of psychological empowerment in connecting environmental stimuli with individual behavioral outcomes. This mediating effect can be explained by COR theory [22], which posits that transition shock acts as a persistent stressor that gradually depletes individuals′ psychological resources, rather than directly impairing competency. In the context of nursing interns, transition shock primarily erodes intrinsic psychological empowerment, specifically compromising its core dimensions of meaning, self‐efficacy, autonomy and impact [26], rather than directly reducing informatics competency itself. Meaning and autonomy are particularly affected: the gap between professional ideals and clinical reality inherent in transition shock undermines interns’ perceived work value [14, 49], while the hierarchical structure, necessary clinical supervision, and limited opportunities for autonomous decision‐making in teaching hospitals may constrain their perceived professional independence [50]. At the same time, self‐efficacy and perceived impact are also weakened: repeated feelings of incompetence when facing unfamiliar and complex tasks may erode confidence in mastering clinical and information system‐related skills [51], while limited participation in patient care decision‐making further reduces their sense of control over the practice environment [52]. When these core psychological resources that sustain learning motivation, cognitive engagement, and active exploration are depleted, interns’ willingness and ability to independently engage with and master complex information systems decline significantly. Therefore, psychological empowerment acts as the key mediating mechanism linking transition shock to suboptimal competency development, rather than functioning as a direct antecedent.

This is consistent with previous research conclusions that psychological empowerment positively predicts positive academic emotions (*β* = 0.64, *p* < 0.001) and learning autonomy (*β* = 0.44, *p* < 0.001) [53]. Therefore, enhancing psychological empowerment may be an effective strategy to buffer the negative association of transition shock and to facilitate the development of informatics competence among nursing interns. Based on this, when conducting nursing informatics training, educators and clinical mentors should consider maintaining psychological resources to be as important as imparting knowledge and skills. A semi‐experimental study on postgraduate medical students confirmed that team‐based learning (TBL) can effectively improve psychological empowerment [54]. Another parallel‐group randomized controlled trial has confirmed that an 8‐week, 12‐session simulation training program significantly improved both psychological empowerment and confidence in managing patient aggression among psychiatric nursing students [55]. This finding suggests that nursing educators can organize similarly structured TBL discussions or simulation training centered on clinical informatics problems to strengthen interns’ professional competence, autonomous decision‐making skills, and psychological empowerment. Notably, the implementation of these strategies, including the frequency and complexity of TBL or simulation modules, must be adapted to the specific context. Consistent with our finding that teaching hospital grade moderates the transition shock–psychological empowerment pathway, interventions should be tailored to fit the unique workflow intensities and supervisory cultures of tertiary and non‐tertiary hospitals, respectively. This tailored approach is essential to effectively buffer against the resource depletion caused by transition shock and to consolidate the psychological foundation required for developing nursing informatics competency. Beyond structured training modules, during clinical internships, there should be a conscious effort to continuously replenish nursing interns′ psychological empowerment by granting progressive decision‐making authority, providing timely positive feedback, and establishing a stable mentor support relationship [56], to counteract the losses caused by transition shock and lay a solid psychological resource for their development of nursing informatics competence.

Further analysis revealed that the grade of teaching hospitals significantly moderated the mediating path of the “transition shock–psychological empowerment–informatics competency” mentioned above. Simple slope analysis indicated that the negative impact of transition shock on psychological empowerment in non‐tertiary‐A teaching hospitals was approximately 2.17 times stronger than that in tertiary‐A teaching hospitals. Compared with non‐tertiary‐A hospitals, the clinical learning environment of tertiary‐A teaching hospitals appeared to more effectively buffer the negative association between transition shock and psychological empowerment, thereby attenuating its indirect negative association with nursing informatics competency. The moderating effect of hospital grades on nursing interns′ psychological empowerment may stem from disparities in resource provision across clinical settings. In Chinese tertiary‐A hospitals, there are more high‐quality supportive learning resources (such as advanced HIS, systematic training, dedicated teaching staff, etc.), forming a rich “environmental resource bank.” A study in Saudi Arabia also confirmed that the educational environment in tertiary referral hospitals positively associates with the learning experiences of medical students [57]. These resources can replenish interns’ depleted psychological resources (such as a sense of meaning and efficacy), thereby safeguarding their learning motivation and promoting the development of their nursing informatics competency. By contrast, in the learning environment of non‐tertiary hospitals with relatively weak support systems and resources, interns are more likely to experience a depletion of psychological empowerment, which disrupts the learning process. Therefore, for nursing interns at non‐tertiary‐A hospitals, targeted interventions, including enhanced preceptorship, simulation‐based training, and TBL, are needed to reduce the psychological burden of transition shock and cultivate psychological empowerment among nursing interns [54, 55]. However, it should be emphasized that the “resource supply” function of teaching hospitals is complex. Even in large teaching hospitals, students′ perceptions of the learning environment are mixed: while they perceive abundant learning opportunities, they may also encounter challenges such as heavy work pressure [58]. Therefore, the core difference lies not in the “tertiary‐A” or “non‐tertiary‐A” labels, but in whether this clinical learning environment can provide an effective protection system that replenishes psychological resources and buffers stress through accessible structural support for nursing interns. Although these findings suggest that tertiary‐A hospital environments play a buffering role, this interpretation remains tentative because of the cross‐sectional design of our study and needs to be verified by future longitudinal research.

## 5. Conclusions

This study constructed and tested a moderated mediating model, revealing the psychological and environmental mechanisms underlying the development of informatics competence among nursing interns. The results indicated that the transition shock experienced by nursing interns weakened their psychological empowerment, thereby indirectly undermining their nursing informatics competency. Moreover, the strength of this pathway was moderated by the teaching hospital grade. Tertiary‐A hospitals could effectively buffer the erosion of psychological empowerment caused by the transition shock, thereby better protecting the psychological foundation for the development of nursing informatics competency. This suggests that the level of nursing informatics competency is not only determined by knowledge and skill training but also by whether nursing interns′ intrinsic psychological empowerment can be protected under career transition pressure. Therefore, the cultivation of nursing informatics competency must be regarded as an integrated process involving the management of psychological resources and environmental support. This study offers an evidence‐based, practical perspective for cultivating informatics competence among nursing interns. Educators and clinical instructors should simultaneously support their learning of knowledge and skills and the maintenance of psychological resources and offer support in accordance with the clinical learning environment.

## 6. Study Limitations

This study has the following limitations: First, all study samples were recruited from a single region, so the external validity of our conclusions across different cultural backgrounds and medical systems requires further verification. Additionally, this study did not record the specific names and number of graded teaching hospitals, which reduces the transparency of the sampling procedure and prevents us from examining heterogeneity across hospitals within the same grade. Second, all core variables were measured via self‐assessment, which may give rise to common method bias and social desirability bias. Third, the cross‐sectional design adopted in this study means we cannot confirm causal relationships between variables, nor can we rule out the interference of reverse causality or unmeasured confounding factors. Fourth, the current model did not control for several covariates that may influence nursing informatics competency, such as prior experience using computers or informatics systems, individual attitudes toward information technology, and the frequency or duration of using HIS during clinical internships. Finally, this study only dichotomized hospital grade into tertiary‐A and non‐tertiary‐A categories and did not further examine the specific composition of internal support factors (e.g., quality of teaching staff, maturity of information systems, and availability of teaching resources) or their respective distinct effects.

Based on these limitations, several directions for future research are proposed. First, future research adopting multicenter and longitudinal designs is needed to validate the temporal relationships among variables and improve the generalizability of findings across diverse cultural and healthcare contexts. Second, future studies can explore the moderating or mediating roles of specific organizational environmental factors (e.g., teaching support, information system accessibility) and individual traits (e.g., prior informatics training, technology attitudes). Third, future research should use more refined institutional indicators, such as teaching evaluation ratings and information system maturity scores, instead of relying solely on the broad classification of “tertiary‐A versus non‐tertiary‐A” hospitals to better capture heterogeneity within the same hospital grade. In addition, measurement accuracy can be improved by incorporating data from multiple sources, such as preceptor evaluations and system operation logs, to complement self‐reported data and reduce potential bias.

## Author Contributions

Miao Wang: writing–original draft and editing, conceptualization, supervision, data analysis, data curation, and funding acquisition.

Xinle Zhou: writing–original draft, conceptualization, investigation, funding acquisition, and data curation.

Tong Wu: investigation.

Xudong Fan: investigation.

Qing Pan: data analysis and methodology.

Hui Guan: investigation and writing–review and editing.

## Funding

This study was supported by the Wannan Medical College’s Teaching Research Project (2024xxkc02) and the National College Students’ Innovation and Entrepreneurship Training Program (202410368040). This work was also supported by the Wannan Medical College Academic and Technical Leaders Reserve Candidates Program and the 2019 Project of the Higher Education Outstanding Young Talents Support Program (Grant No. gxyqZD2019039).

## Ethics Statement

The study was approved by the Research Ethics Committee of the School of Nursing at Wannan Medical College, China (NO. 20250010). The study was conducted in compliance with the principles of the Declaration of Helsinki. Before enrollment, all participants received detailed information regarding the study’s purpose, procedures, potential risks, benefits, confidentiality, and the voluntary nature of participation. Written informed consent was obtained from all participants, and the study adhered to applicable ethical standards.

## Conflicts of Interest

The authors declare no conflicts of interest.

## Data Availability

The datasets used and/or analyzed in this study are available from the corresponding author upon reasonable request.
